# Quantitative Assessment of Point-of-Care 3D-Printed Patient-Specific Polyetheretherketone (PEEK) Cranial Implants

**DOI:** 10.3390/ijms22168521

**Published:** 2021-08-07

**Authors:** Neha Sharma, Soheila Aghlmandi, Federico Dalcanale, Daniel Seiler, Hans-Florian Zeilhofer, Philipp Honigmann, Florian M. Thieringer

**Affiliations:** 1 Clinic of Oral and Cranio-Maxillofacial Surgery, University Hospital Basel, CH-4031 Basel, Switzerland; neha.sharma@usb.ch (N.S.); hans-florian.zeilhofer@usb.ch (H.-F.Z.); 2 Medical Additive Manufacturing Research Group (Swiss MAM), Department of Biomedical Engineering, University of Basel, CH-4123 Allschwil, Switzerland; philipp.honigmann@ksbl.ch; 3 Basel Institute for Clinical Epidemiology and Biostatistics, Department of Clinical Research, University Hospital Basel, CH-4031 Basel, Switzerland; soheila.aghlmandi@usb.ch; 4 Institute for Medical Engineering and Medical Informatics, University of Applied Sciences and Arts North-Western Switzerland, CH-4132 Muttenz, Switzerland; federico.dalcanale@fhnw.ch (F.D.); daniel.seiler@fhnw.ch (D.S.); 5 Hand Surgery, Cantonal Hospital Baselland, CH-4410 Liestal, Switzerland; 6 Amsterdam UMC, Department of Biomedical Engineering and Physics, University of Amsterdam, Amsterdam Movement Sciences, NL-1105 Amsterdam, The Netherlands

**Keywords:** alloplastic implant, biocompatible material, computer-assisted, cranioplasty, fused filament fabrication, reconstructive surgery, patient-specific modeling, printing, polymer, three-dimensional

## Abstract

Recent advancements in medical imaging, virtual surgical planning (VSP), and three-dimensional (3D) printing have potentially changed how today’s craniomaxillofacial surgeons use patient information for customized treatments. Over the years, polyetheretherketone (PEEK) has emerged as the biomaterial of choice to reconstruct craniofacial defects. With advancements in additive manufacturing (AM) systems, prospects for the point-of-care (POC) 3D printing of PEEK patient-specific implants (PSIs) have emerged. Consequently, investigating the clinical reliability of POC-manufactured PEEK implants has become a necessary endeavor. Therefore, this paper aims to provide a quantitative assessment of POC-manufactured, 3D-printed PEEK PSIs for cranial reconstruction through characterization of the geometrical, morphological, and biomechanical aspects of the in-hospital 3D-printed PEEK cranial implants. The study results revealed that the printed customized cranial implants had high dimensional accuracy and repeatability, displaying clinically acceptable morphologic similarity concerning fit and contours continuity. From a biomechanical standpoint, it was noticed that the tested implants had variable peak load values with discrete fracture patterns and failed at a mean (SD) peak load of 798.38 ± 211.45 N. In conclusion, the results of this preclinical study are in line with cranial implant expectations; however, specific attributes have scope for further improvements.

## 1. Introduction

Large cranial bone defects can result from decompressive craniectomy following cerebral infections, head trauma, or resection of bone-invading intracranial tumors. Cranioplasty is a surgical reconstructive procedure that re-establishes the physiological functions of the neurocranium and restores the structural integrity of cranial defects [1,2]. The goal of cranioplasty is not only restitution of the cranial defect, but also to improve the esthetics, quality of life, and psychological wellbeing of the patient [3,4].

With advances in computer-aided design (CAD) and computer-aided manufacturing (CAM) technologies, cranial reconstructions have witnessed tremendous progress [5,6,7,8,9,10]. In particular, additive manufacturing (AM) or three-dimensional (3D) printing have become ways of aptly reconstructing the patient’s affected surgical anatomy with patient-matched implants [11,12,13,14]. Patient-specific implants (PSIs), in general, are driven by the imperative need of surgeons to treat complicated reconstructive cases that demand a unique patient-specific approach [15,16,17]. The potential use of 3D printing in these realms as a source of customizable PSIs that matches each patient’s unique anatomy has piqued great interest among surgeons.

There have been several reports on applying point-of-care (POC) 3D printing in numerous avenues, including the fabrication of anatomical biomodels for preoperative surgical planning, surgical guides, and prosthetic aids [18,19]. Particularly in the context of cranioplasty, recently published reports have demonstrated the fabrication of customized acrylic cranioplasty implants in hospitals assisted by 3D-printed molds [20,21,22,23]. However, very few reports have addressed the avenue of POC manufacturing of 3D-printed personalized implants.

Material extrusion-based 3D printing from thermoplastic polymer filaments usually referred to as fused filament fabrication (FFF), is the most commonly used AM technique in hospitals due to its ease of operability and availability of low-cost machines. However, FFF technology has been limited to the production of anatomical biomodels and has not yet been adopted into the mainstream production of functional implants [24,25,26]. With advancements in AM systems, 3D printing of high-temperature thermoplastic polymers such as polyetheretherketone (PEEK) and prospects for customized FFF 3D-printed PEEK surgical implants have emerged, increasing attention for POC manufacturing [27,28,29]. Although milled PEEK implants have been used in cranial reconstructions for a long time [7,17,30], the commercial formulation of medical-grade PEEK filaments for extrusion-based 3D printing is relatively recent. By definition, any innovative technology is exploratory, and guidelines for using these novel approaches are needed to reduce the likelihood of errors [31].

Consequently, investigating the clinical reliability of POC-manufactured implants in the preclinical phase has become a necessary endeavor. Our previous studies have demonstrated the feasibility of using high-temperature FFF 3D printers explicitly tailored to produce customized PEEK implants in hospitals [32,33]. However, this augmented employment of 3D printing opens up questions regarding effectiveness and safety in the use of POC-manufactured PEEK biomedical implants.

Therefore, this paper aims to provide a quantitative assessment of the potential clinical efficacy of POC-manufactured 3D-printed PEEK PSIs for cranial reconstruction. In addition to the description of the technical digital workflow—from virtual surgical planning (VSP) to material extrusion-based 3D printing of these PSIs—the objectives of this work are the presentation and characterization of the geometrical, morphological, and biomechanical aspects of the FFF 3D-printed PEEK cranial implants for their use as well as to reveal the difficulties which may occur in clinical application.

## 2. Results

### 2.1. Geometric Characteristics of the FFF 3D-Printed PEEK Patient-Specific Cranial Implants

**Figure 1:**
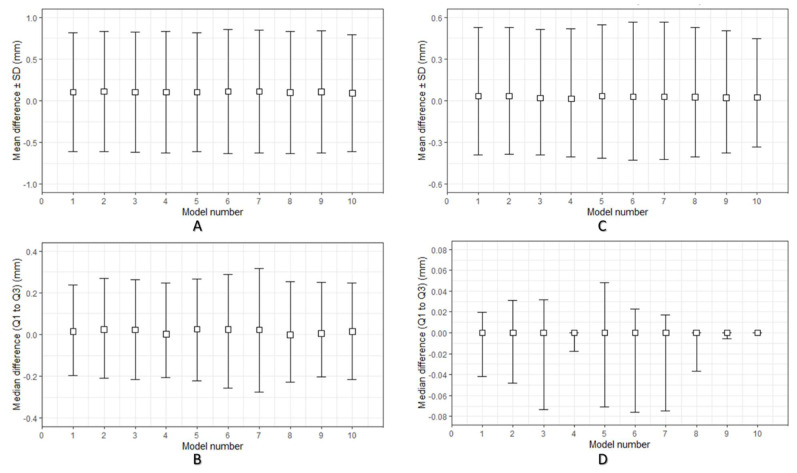
Descriptive data distribution illustrating the geometric characteristics of the material extrusion 3D-printed PEEK cranial patient-specific implants (specimen model 1–10): (**A**) Dimensional accuracy mean difference ± standard deviation (SD) (mm) between planned and 3D-printed PEEK cranial implants; (**B**) Dimensional accuracy median difference (Q1 to Q3) (mm) between planned and 3D-printed PEEK cranial implants; (**C**) Dimensional repeatability mean difference ± standard deviation (SD) (mm) among 3D-printed PEEK cranial implants; (**D**) Dimensional repeatability median difference (Q1 to Q3) (mm) among 3D-printed PEEK cranial implants.

Figure 1A,B illustrate the overall descriptive data distribution of the deviation analysis for dimensional accuracy between planned (reference) and FFF 3D-printed PEEK PSIs. For quantitative assessment of the dimensional accuracy of the 3D-printed PEEK PSIs, RMSE values were analyzed. The comparative analyses revealed a mean RMSE ± SD value of 0.731 ± 0.013 mm, whereas the median (Q1 to Q3) RMSE value was 0.733 (0.723 to 0.739) mm.

Figure 1C,D illustrate the overall descriptive data distribution of the deviation analysis for dimensional repeatability in FFF 3D-printed PEEK PSIs. For quantitative assessment of the dimensional repeatability of the printed PSIs, RMSE values were analyzed. The comparative analyses revealed an overall mean RMSE ± SD value of 0.155 ± 0.081 mm, whereas the median (Q1 to Q3) RMSE value was 0.120 (0.093 to 0.231) mm. A summary of all statistics concerning RMSE values for dimensional repeatability analysis is illustrated in Table 1.

In the dimensional repeatability analysis, the Shapiro–Wilk test yielded significant results. Combined with the visual inspection of the Q–Q (quantile–quantile) normal plot, the results displayed a non-normality distribution of data. Therefore, a non-parametric statistical test was carried out to identify intergroup differences. The pairwise comparison using Tukey’s post hoc test revealed a statistically significant difference (*p* < 0.05) between cranial implant 8 and cranial implant 7 (Table 2).

### 2.2. Morphological Characteristics of Anatomical Reconstructions with 3D-Printed PEEK Patient-Specific Cranial Implants

For morphological assessment, the symmetry of 3D CAD cranial reconstructions was examined. The CAD reconstruction results revealed excellent cranial reconstruction symmetry with a mean RSI ± SD value of 97.48 ± 0.023% and a median (Q1 to Q3) RSI value of 97.47% (97.47% to 97.49%).

**Figure 2:**
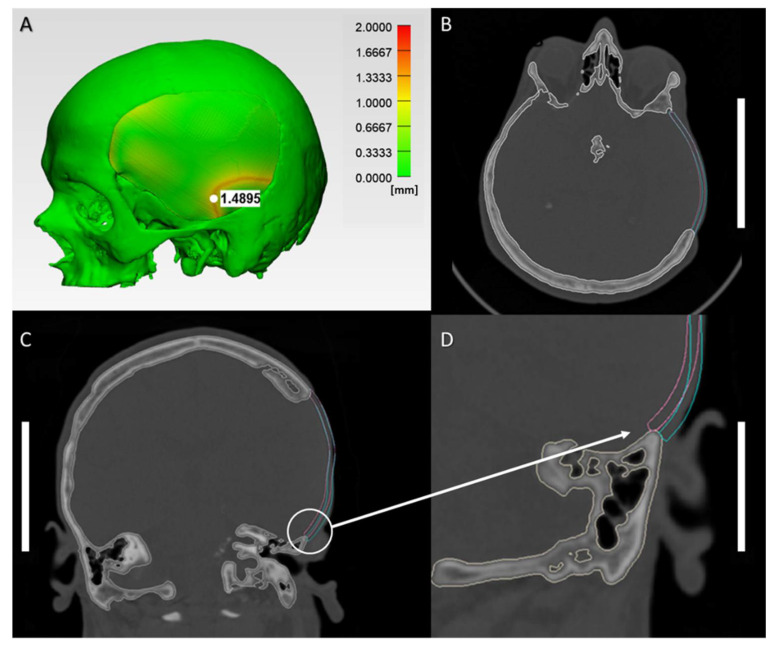
Morphological characteristics in an implant with a “satisfactory” fit and contour continuity: (**A**) Reconstruction contour conformance distance map; (**B**) Axial view illustrating the contour continuity between planned (blue) and 3D-printed (pink) PEEK implant (scale bar, 5 cm); (**C**) Coronal view illustrating the contour continuity between planned (blue) and 3D-printed (pink) PEEK implant (scale bar, 5 cm); (**D**) Magnified sagittal cross-sectional view of the white circled inset illustrating the slight discrepancy in the marginal fit between planned (blue), and 3D-printed (pink) PEEK implant (scale bar, 5 cm).

The morphological similarity was assessed with reconstruction contour conformance distance mapping. The results show close conformance between the 3D CAD cranial reconstruction with printed PSI and the planned reconstruction. The quantitative comparative analyses revealed an overall mean RMSE ± SD value of 0.499 ± 0.032 mm, and a median (Q1 to Q3) RMSE value of 0.499 (0.494 to 0.514) mm. The morphological fit and contour continuity of the 3D-printed PSIs on the cranial defect model were mainly rated as “good” (6; 60%) or “satisfactory” (4; 40%). Figure 2A demonstrates the conformance distance map for 3D CAD cranial reconstruction with printed PEEK PSI in an exemplary case with a “satisfactory” morphological fit. The regions of low similarity are shown in red, whereas areas in green indicate the opposite. The overall conformance distance was approximately under 2 mm over most of the surface area. Figure 2B,C illustrate the qualitative assessment for the “satisfactory” morphological fit and contour continuity between the printed PEEK cranial implants with the 3D CAD cranial defect model in axial and coronal cross-sectional views. The fit and contour continuity was maintained at the anterior margin near the frontotemporal region; however, a slight deformation altered the implant’s tangential fit at the posterior margin near the infratemporal region, as depicted in the magnified sagittal cross-sectional view (Figure 2D). This slight change in the conformance distance (≈ 1.5 mm) transformed the implant shape from an onlay-fitting to an inlay-fitting implant at the infratemporal region.

### 2.3. Biomechanical Characteristics of the 3D-Printed PEEK Patient-Specific Cranial Implants

The findings of the quasi-static mechanical test are presented as force–displacement curves and as a post-test examination of the cranial implant specimens. Overall, the FFF 3D-printed customized PEEK cranial implants had a mean peak force of 798.38 ± 211.45 N and occurred on an average displacement of 2.54 ± 0.56 mm. Table 3 illustrates the peak force and respective displacement at peak force noticed within each 3D-printed customized PEEK cranial implant.

**Figure 3:**
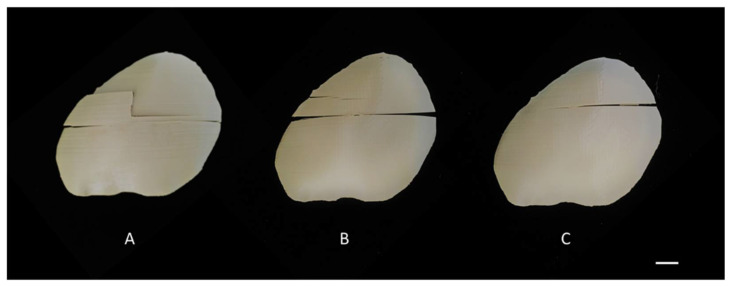
Different fracture patterns in the material extrusion 3D-printed PEEK cranial patient-specific implants: (**A**) Class 1 (scale bar, 1 cm); (**B**) Class 2 (scale bar, 1 cm); (**C**) Class 3 (scale bar, 1 cm).

We observed differences in the damage pattern configurations of implants with an indication for class 1 (4; 40%), class 2 (4; 40%), and class 3 (2; 20%) fracture patterns (Figure 3). The implants with class 1 (Figure 3A) fracture pattern had the highest overall peak force (>800 N), followed by class 2 (Figure 3B) fracture pattern implants (600–800 N), whereas the lowest peak force was seen in class 3 (Figure 3C) fracture pattern implants (<600 N). It was noted that the PSIs with a “satisfactory” morphological fit and contour continuity had a class 2 fracture pattern. In contrast, the PSIs with a “good” fit had a distribution between class 1 and class 3 fracture patterns.

**Figure 4:**
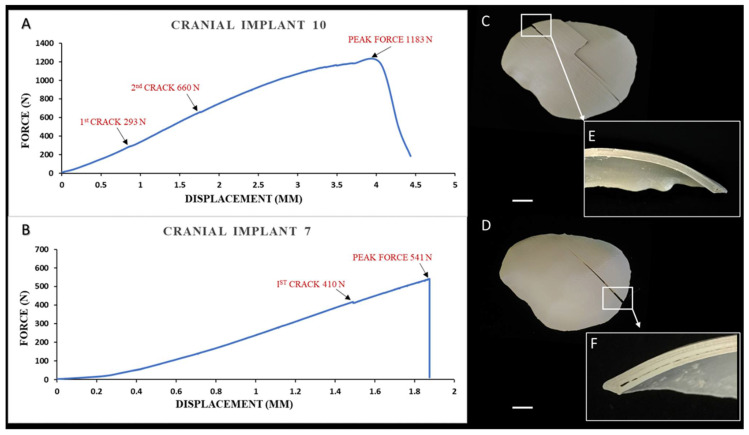
Biomechanical characteristics of the material extrusion 3D-printed PEEK patient-specific cranial implants: (**A**) Force–displacement curve response in an implant with the highest peak load; (**B**) Force–displacement curve response in an implant with the lowest peak load; (**C**) Multi-layered class 1 fracture pattern with intra- and interlayer connectivity (scale bar, 1 cm); (**D**) Linear class 3 fracture pattern confined to the intralayer interface (scale bar, 1 cm); (**E**) Magnified view of the white inset box displaying strong interlayer bonding in cranial implant 10; (**F**) Magnified view of the white inset box illustrating weak interlayer bonding with visible air gaps in cranial implant 7.

The force–displacement curve responses for the strongest (class 1) and weakest performing (class 3) representative cranial implants are shown in Figure 4A; a typical class 1 response obtained from the uniaxial compressive test is reported in Figure 4A. In particular, the figure shows the force and deflection acquired during the test with a plateauing force, referred to as peak force seen at 1183 N, and a shift from elastic to plastic deformation was noted. The first microcrack initiation was around 293 N, with a second crack propagation around 660 N (Figure 4A). The post-peak region illustrated a macrocracking semi-brittle type of failure. On post-test examination, it was observed that the shape of the cranial implant was retained, and the fracture pattern was confined to the printed intra- and interlayer interfaces (Figure 4C). The cross-section of the cranial specimen (Figure 4E) exhibited a strong interlayer bonding after the implant specimen was broken.

In contrast, in the weakest implant case scenario (cranial implant 7), the peak force was noticed at 541 N, with the first crack initiation at around 410 N (Figure 4B). The post-peak region depicted a macrocracking brittle-type failure with no plastic deformation. At the post-test examination, a linear fracture pattern confined to the intralayer interface with air gaps was observed (Figure 4D), and the cranial implant specimen displayed weak interlayer bonding strength, as shown in Figure 4F.

## 3. Discussion

Over the years, PEEK has emerged as the biomaterial of choice to reconstruct craniofacial defects [7,17,34,35,36,37]; however, little is known about FFF 3D-printed PEEK implants. With advances in 3D printing technology, prospects for customized FFF-printed PEEK surgical implants have emerged [27,33,34]. The current literature relating to the geometric and morphological characteristics of in-hospital 3D-printed PEEK cranial PSIs is very limited. Although few studies have evaluated the accuracy of cranial reconstruction with PSIs fabricated in-hospital, these studies are primarily directed towards molded acrylic prostheses [20,22,38,39,40]. One study focused on quantitative assessment of the dimensional accuracy of patient-specific laser-sintered PEEK cranial implants concerning the build orientation [41]. The authors stated that transversally (horizontal) manufactured implants exhibited the slightest deviation from the planned model. Our previous study [33] showed that the transverse orientation of implants in FFF PEEK printing results in a sub-optimal surface finish of the implants, which is clinically unacceptable. Therefore, considering the working principle of FFF, the implants in the present study were fabricated in an axial orientation with minimal generation of support structures. According to our results, FFF 3D-printed PEEK PSIs were within an acceptable dimensional accuracy range required in cranioplasty reconstructive procedures, with overall deviations under 1.0 mm. Furthermore, the dimensional precision of the FFF system revealed that the fabricated PEEK cranial implants had high reproducibility, with overall deviations under 0.5 mm. Even though statistically significant differences were depicted between cranial implant 8 and cranial implant 7, the difference was less than 0.2 mm and, therefore, was clinically irrelevant.

The morphological characterization of the FFF 3D-printed PEEK PSIs is essential for estimating the potential cranial reconstruction accuracy. Therefore, further analysis of the spatial distribution of variations concerning the morphological cranial (anatomical) reconstruction was performed. We applied the symmetrical index method [42] to assess the symmetry of the 3D cranial reconstruction with FFF 3D-printed PEEK implants. Basu et al. [39] and Tan et al. [43] reported the reconstruction symmetry in acrylic cranioplasty with mean values of 94.5% and 96.2%. The symmetry achieved in our study was excellent, with a mean RSI value of 97.5%. The preclinical results potentially establish the clinically acceptable restoration of skull defects that can be achieved with intraoperative FFF 3D-printed PEEK cranial implants.

The morphological conformance of PSIs with cranial defect components is a crucial element and is often underestimated in clinical studies. Discrepancies noticed in the morphological fit of the implant onto the bony component can result in postoperative complications. Few researchers have presented an objective volumetric assessment of the implant fit with dice similarity coefficient (DSC) and utilizing a matrix-based assessment to compare implant gaps with native bone at different anatomic regions in postoperative images [40,44]. Our preclinical results present a colorimetric surface-deviation conformance map established on a numerical index—the RMSE, illustrating point-based as well as overall conformance distance deviations. Quantitatively, the co-registered cranial reconstructions showed an overall conformance distance under 1.0 mm throughout most surface areas. Qualitatively, most of the PSIs revealed “good” and a definite morphologic similarity concerning fit and contour continuity, translating into a PSI that effectively restores the cranial anatomical contours with no intraoperative modification. However, some PSIs displayed a “satisfactory” morphologic similarity, which translates into a PSI that effectively restores the cranial anatomical contours requiring minor intraoperative modification. The point-based analysis in these PSIs further revealed that most of the differences were the lowest (highest congruency) in the squamous area and the highest (lowest congruency) in the infratemporal area. These slight temporal region discrepancies can always be camouflaged by overlying soft tissue. The primary value of this analysis is the quantitative confirmation of our preclinical experience that the FFF 3D-printed PEEK PSIs provide a close morphologic approximation of the cranial defect.

Clinical studies about individual cases or case series for cranial PSIs often overlook detailed reporting of the design specifications [7,17]. This could partly be because the fabrication process is outsourced to external service providers and is of relative interest to the surgical team. However, design considerations are a critical need for surgeons embarking on the POC manufacturing of customized implants. Design for additive manufacturing (DfAM) requires expertise in several areas, particularly concerning design specifications, optimized build orientations, support structures, fixation points, and clinically acceptable tolerance levels of the fabricated implant. In the context of non-load-bearing patient-specific PEEK cranial implants, the size, asymmetrical shape, thickness, contour, and edge profile are the key design elements for consideration. The main (base) cranial implant (typically 3–5 mm thick) is without fixation tabs and can be attached to the cranial bone with any commercially available fixation systems (miniplates) to provide a precise fit. The next design element is the edge profile, categorized into an inlay (drop-in-fit) or an onlay (overlapping margins resting over the bone defect) fitting implant. Unlike inlay design, the onlay edge profile fit provides intraoperative flexibility if minor adjustments and alterations of bony margins are needed. Perfusion holes (typically 1.5–2 mm in diameter, spaced 10–25 mm apart) are the design elements that allow the passage of fluid, the attachment of temporalis muscle, and dura retention. In FFF PEEK 3D printing, perfusion holes need to be strategically placed because plate fractures might occur during the printing process [33], or hole reaming after printing can be a solution.

Another aspect that needs consideration is the integrated fixation system—a design element used to fixate the main implant to the cranial bone. The fixation tabs are typically 4–6 extensions on the base cranial plate design. These extensions can be further prolonged to accumulate larger-than-estimated surgical resections. The printing of edge profile extensions can be challenging in FFF PEEK 3D printing, resulting in a suboptimal surface finish and stemming post-processing steps. An alternative is to use overlapping margin fits with angular screw fixation points to provide a more rigid fixation. Furthermore, the fixation over the infratemporal region depends on the cranial defect size and surgical exposure needed. In addition, a temporal cutback (an anatomical free space between the bony defect margin and caudal margin of the implant) is a design element that dictates the shape of the base implant in the temporal region of the skull to aid in reducing intraoperative tissue exposure or temporalis muscle damage during surgery. Due to the inherent printing mechanism of FFF, another aspect that needs to be considered is the support structures. If printed as one unit, the fabrication of cranial implants for defects crossing the midline or with orbital involvement needs extensive support structures. These support structures contribute to many post-processing steps. Therefore, strategies to minimize post-processing procedures by altering design and manufacturing strategies can be beneficial. Finally, a multi-part cranial implant—is a beneficial design element if the implant’s geometry has complex contours and curvatures, such as large-sized cranial defects crossing the midline or cranial defects with a lateral orbital wall or orbital roof involvement. This approach is favorable from the FFF PEEK manufacturing point of view and provides insertion-path autonomy to implant components.

It should be noted that the overall quality of a POC-manufactured PSI is based not only on its fit, margins, and contours, but also on its function. For this reason, it is pertinent to assess the biomechanical properties of an implant and evaluate whether the implantation’s structural integrity is suitable for implantation. Our results demonstrated the load-bearing capacity of FFF 3D-printed PEEK cranial PSIs in a quasi-static test setup. The implants used in this study were of the same form as those used clinically in patient-specific or customized implants. We noticed that the implants failed at a mean (SD) peak load of 798.38 ± 211.45 N. Motherway et al. [45] experimentally determined the strength for parietal cranial bone in a quasi-static test setup and reported a maximum load of 793.7 N. Our result for the maximal load seems to be in line with the experimentally determined parietal cranial bone data. However, it was noticed that the tested implants in our study had differing peak force values with discrete fracture patterns and were inconsistent when compared with each other. This can be partly explained due to the difference in the interlayer bonding strength of FFF 3D-printed PEEK samples. The implants with the highest peak load had a strong bonding with uniform PEEK fusion and interlayer connectivity, whereas air gaps and infill fusion lines were observed in implants with the lowest strength. It was evident that in FFF 3D-printed PEEK PSIs, the process of an early stage of fracture was highly reliant on void coalescence. A crack initiation area was observed in the major fracture patterns, closely followed by a striation area. With increasing load, the striations tended to become closer as the distance from the crack initiation zone increased. Finally, a transition to rapid crack propagation occurred, eventually breaking the implant. Unlike conventionally available CAD/CAM milled and laser-sintered PEEK implants [41,46], the FFF 3D-printed PEEK PSIs in our study retained their shape after structural failure without multi-fragmentation. From a clinical standpoint, this failure pattern (non-fragmentation behavior) was favored in failed implant retrieval.

In FFF PEEK printing, thermal processing conditions and printing parameters play a crucial role. Optimum thermal management and heat distribution around the printed part is essential to reduce the residual stress and increase the print quality [33,47,48]. When a larger layer height (above 0.2 mm) is selected, void formation might occur in the samples, resulting in weak interlayer bonding. Vaezi and Yang [47] stated that air gap formation is an inherent limitation of the FFF PEEK printing process. Air gaps tend to be formed either due to the PEEK filament’s geometry, limited deposition of the material, especially at the perimeters in complex geometries, and temporary variation in the extrusion rate. Additionally, micro-air bubbles can be entrapped inside the feedstock filament due to moisture, leading to void formation. All these defects induce porosity into FFF 3D-printed PEEK parts, consequently leading to decreased mechanical properties. To mitigate these issues, it is essential to implement an integrated system within the FFF PEEK printer that keeps the filament dry during the printing process to avoid entrapped micro-air bubble formation. In addition, especially in large-sized prints such as cranial implants, a new and cleaned nozzle should be used for every print to avoid temporary extrusion issues. Furthermore, support structure optimization surrounding the implant should be considered for continuous perimeter filling and optimal filament bonding.

Several authors have assessed the biomechanical properties of curvilinear-shaped cranial implants using a quasi-static test setup, as outlined in Table S1. Two studies evaluated the structural integrity of selective laser-sintered (SLS) 3D-printed porous PEEK cranial implants. Depending on cranial implant design and printing process parameters, peak loads around 600–1000 N were determined [41,49]. Lethaus et al. [46] tested a solid CAD/CAM milled PEEK cranial implant and reported a failure at a peak load of 24 kN. The authors also tested a solid titanium cranial implant in the same study, and reported that no implant damage was noticed at a peak load of 50 kN. However, it should be noted that the solid implants tested in their study were considerably thicker (6 mm) than those typically utilized in clinical cases. Ono et al. [50] assessed the structural integrity of porous hydroxyapatite cranial implants and reported that the implants failed at a peak load of 165 N. Another study tested porous hydroxyapatite implants covered with silicone rubber, and a peak load of 726 ± 345 N was detected [51]. On the other hand, the strength of bioactive glass-fiber-reinforced composite cranial implants was reported at around 175 ± 101 N [52]. In composite material, especially titanium-reinforced calcium phosphate cranial implants, peak loads in the range of 457–808 N have been reported [53,54,55].

There is a lack of consensus and standardized guidelines capable of providing research protocols for mechanical characterization when it comes to cranial implants. This lack is most likely because high mechanical loads are seldom applied to the skull bone; thus, cranial implants are considered non-load-bearing implants. Moreover, due to differences in test specimen shape, geometry, and indenter shape, inter-study comparison becomes incomprehensible. Nonetheless, another significant clinically applicable criterion was that the requirement for an initial strength value of at least 20 kg before and immediately after surgery could be fulfilled [50]. Based on clinical practice, we believe that an initial strength value of up to 20 kg (196 N) is the bare minimum strength of implants for cranial reconstruction.

The study limitations include simplification of the test setup. The force applied to the implant was quasi-static, which may not accurately reflect the loading condition in a real-life scenario when an external impact force is applied to the implant. Secondly, it would be interesting to evaluate the fracture pattern in non-fixated and screw-fixated FFF 3D-printed PEEK implants in future studies. Thirdly, there was a lack of quantitative analysis on the internal configuration of the printed cranial implants. Further studies involving non-invasive, non-destructive inspection of the printed cranial implants could help to precisely investigate internal porosity induced due to the printing process.

## 4. Materials and Methods

**Figure 5:**
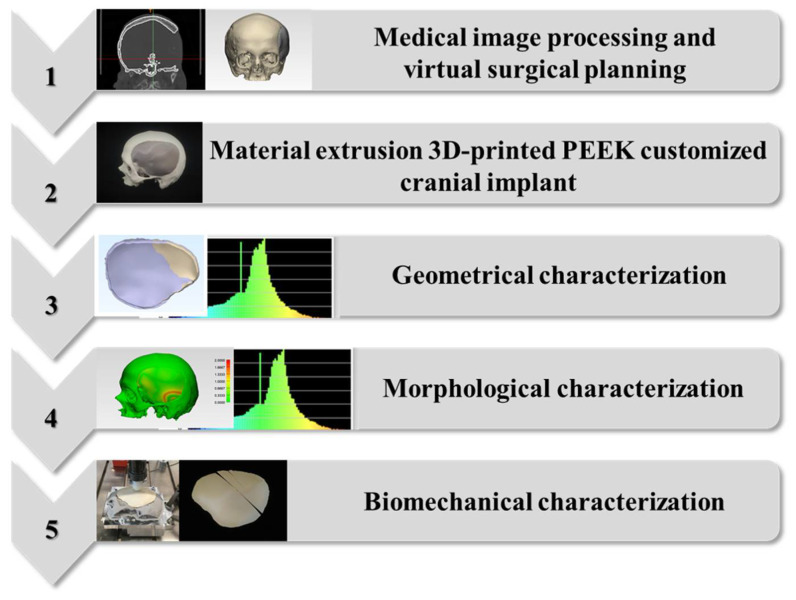
Graphical flowchart summarizing the study workflow.

The study workflow consisted of the following five protocols: (1) medical image processing and virtual surgical planning; (2) material extrusion-based FFF 3D printing of PEEK PSIs; (3) geometrical characterization of FFF 3D-printed PEEK PSIs; (4) morphological characterization of the cranial reconstruction with FFF 3D-printed PEEK PSIs; and (5) biomechanical characterization of FFF 3D-printed PEEK PSIs. Figure 5 illustrates a graphical flowchart summarizing the study workflow.

### 4.1. Medical Image Processing and Virtual Surgical Planning (VSP) Protocol

An anonymized unilateral cranial defect case was selected from the University Hospital Basel database for this study. We selected this case because it was a representative defect in an average craniotomy. The chosen case was then categorized based on a classification system of cranial implants, which considered the anatomical location and the degree of difficulty in designing and manufacturing the implant [56]. The case was a class III (fronto–temporo–parietal) cranial defect, demonstrating a unilateral defect with a size larger than 100 cm^2^. When designing cranial implants, it is crucial to consider the complicated geometry of the defects. The size of cranial defect has implications on the implant’s design and manufacturing process—the larger the defect, the larger the span of curvature that has to be reconstructed.

The detailed workflow for medical image acquisition, segmentation, and anatomical modeling of customized cranial implants has been described in our previous studies [33,57]. The image acquisition parameters selected for a computed tomography (CT) (Siemens SOMATOM, Siemens Healthcare GmbH, Erlangen, Germany) scan were: (1) gantry tilt, 0°; (2) slice thickness, 1 mm; (3) seed per rotation, 1 mm; (4) matrix of 512 × 512 pixels, with a pixel size of 0.48 mm; and (5) a high-resolution bone reconstruction algorithm. Briefly, CT datasets (exported in Digital Imaging and Communications in Medicine, DICOM format) were processed by the surgeon using MIMICS Innovation Suite software v. 22.0 (Materialise, Leuven, Belgium). This software allowed us to segment the skull with a semi-automatic algorithm based on bone-specific Hounsfield units (HU). The 3D skull volumetric reconstruction was saved, and the project was imported into CAD software (3-matic medical v. 14.0, Materialise, Leuven, Belgium). Based on the 3D skull model, a mirrored model was created along the midplane defined by anatomically symmetric data points. Subsequently, using a context-driven surface reconstruction algorithm, an optimally shaped cranial plate was generated by interpolating the defect outline, and reconstruction splines designed on intersecting sketches. The CAD file of the designed cranial implant was finally converted and saved in a standard tessellation language (STL) file format.

### 4.2. Material Extrusion 3D Printing Protocol of PEEK Patient-Specific Cranial Implants

A material extrusion, FFF desktop 3D printer developed explicitly for PEEK medical additive manufacturing (Apium M220, Apium Additive Technologies GmbH, Karlsruhe, Germany) was used to fabricate the patient-specific cranial implant. The PEEK 3D printer is intended to produce PSIs in a cleanroom or a hospital environment under the International Organization for Standardization (ISO) 10993 series of standards for the biological evaluation of medical series [58]. The printer has an advanced temperature management system that provides an enclosed heated envelope around the part, with an incremental layer-by-layer airflow temperature build-up during the fabrication process. The cranial PSIs were fabricated in a medical-grade 1.75 mm diameter PEEK filament (Evonik Vestakeep^®^i4 3DF, Evonik Industries AG, Essen, Germany). The filament was extruded from Vestakeep^®^ i4 G resin (Evonik Industries AG, Essen, Germany). This certified implant-grade material meets the requirement of the American Society for Testing and Materials (ASTM) F2026–17 guideline for PEEK surgical implant applications [59].

**Figure 6:**
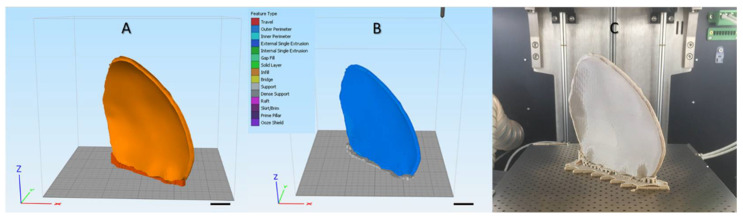
Schematic representation of the steps involved in the material extrusion fabrication process of PEEK patient-specific cranial implants: (**A**) Orientation of the implant in the slicing software on the printer’s build platform with support structures (scale bar, 1 cm) (**B**) G-code generation with the respective optimal printing parameters (scale bar, 1 cm); (**C**) Material extrusion 3D-printed PEEK cranial implant (in situ).

Before fabrication, quality control measures were undertaken through the elimination of errors in the STL file of the cranial implant. This ensured that errors such as intersecting triangles, bad contours, and overlapping edges were resolved (3-matic medical v. 14.0, Materialise, Leuven, Belgium). For fabrication, the STL file of the cranial implant was imported into slicing software (Simplify 3D version 4.0, Cincinnati, OH, USA) to further process and determine the best orientation for fabrication. A 0° orientation (vertical) to the build platform direction (Z-axis) was selected, and support structures were generated on the overhanging structures of the cranial implant (Figure 6A). The printing parameters chosen for the fabrication process are listed in Table 4. The generated g-code file (Figure 6B) with the respective printing parameters was then transferred to the 3D printer software (Apium Print Control v. 3.4.4, Apium Additive Technologies GmbH, Karlsruhe, Germany).

The physical production process consisted of the following quality control steps: (1) humidity level check of the PEEK filament; (2) print bed calibration by a manual bed-leveling procedure; (3) calibration of the FFF system for the extrusion multiplier value. To prevent warping of the implant during printing, automatic raft generation functionality integrated into the 3D printer’s software was enabled. Subsequently, the printing process was initiated, and the fabrication of PEEK cranial PSIs (*n* = 10) was completed. To ensure reproducibility, each PSI was manufactured individually in the center of the build platform for uniform heat distribution (Figure 6C). After printing, the implants were manually separated from the printed raft. The raft was then manually cut from the printer’s build platform. The temporary support material was manually removed using cutting pliers, and scuff marks were then trimmed off the implant’s surface with high-speed rotary burr tools. The implants were not post-processed further.

### 4.3. Geometrical Characterization Protocol for the 3D-Printed PEEK Patient-Specific Cranial Implants

Geometrical characterization of the POC-fabricated implants represents an essential aspect and consists of a combination of dimensional accuracy (trueness) and dimensional repeatability (precision). The geometric characteristics were analyzed according to ISO 5725-2 guidelines [60]. The dimensional accuracy is defined as the closeness of an object’s measured value to a known expected value. In contrast, dimensional repeatability is the closeness of the results in repetitively printed objects [61].

The FFF 3D-printed PEEK cranial PSIs were digitized with an optical scanning system (EinScan-SP, SHINING 3D Tech. Co., Ltd., Hangzhou, China), employing reverse-engineering principles. The scanner utilizes a structured white-light technology and has an accuracy of ≤0.05 mm (EinScan-S series software v. 3.1.0.1). A non-textured, colorless, high-resolution unwatertight scan model of the respective implants was created using the turntable marker alignment functionality. The 3D coordinates digitization process was automatically registered to generate a point cloud of the implant. No further post-processing steps, i.e., smoothing, auto-fill, mesh repair, sharpness filters, were applied. The resultant 3D point cloud data of the implants were consequently converted into STL file format utilizing an automated triangulation algorithm. The digitized STL files of the printed PEEK cranial PSIs were superimposed with the STL file of the planned (reference) cranial prosthesis. Point-to-point (n-point) registration was used to approximately align the two dataset entities. The alignment was further refined in global registration using an iterative closest point (ICP) algorithm. A 3D part-comparison analysis was performed (3-matic medical v. 14.0, Materialise, Leuven, Belgium). The deviations were automatically calculated considering the Euclidean distances between the triangles of the 3D surface meshes. The overall deviations were quantified using the root mean square error (RMSE) values in millimeters for each comparison. The RMSE is a measurement indicator for determining the resemblance between two data groups of n-dimensional vector sets at the same coordinate system. A low RMSE value correlates with high geometric characteristics.

For the assessment of dimensional accuracy, the STL files of the FFF 3D-printed PEEK cranial implants (*n* = 10) were compared with the STL file of planned (reference) cranial implants, and for dimensional repeatability assessments, the STL files of the FFF 3D-printed PEEK cranial implants were compared (*n* = 90).

### 4.4. Morphological Characterization Protocol for the Anatomical Reconstruction with 3D-Printed PEEK Patient-Specific Cranial Implants

To evaluate the morphological characteristics, assessments of the symmetry and conformance mapping of the 3D CAD cranial (anatomical) reconstructions were performed. Computer-assisted symmetry assessments were computed using a reconstruction symmetry index (RSI), expressed as a percentage. A perfectly symmetrical reconstruction would yield an RSI of 100%, whereas a score of 95% or more indicates symmetry [62]. The RSI was calculated using the overlap between the 3D CAD cranial reconstruction with the printed (digitized) PSI to the target (planned) reconstruction. A mirrored model was created based on the 3D volumetric reconstruction of the cranial defect model (3-matic medical 14.0, Materialise, Leuven, Belgium). A midplane or axis of symmetry was used, which replicated the corresponding contralateral healthy (non-defect) anatomical cranial region, resulting in a 100% symmetrical 3D CAD cranial reconstruction. The 3D CAD cranial reconstruction with printed PSI was then superimposed onto the perfect cranial reconstruction, and the area of overlap was obtained. An RSI value was then calculated using the following equation:

RSI = O/T,(1)

where O is the total area of overlap, and T is the total area of the 3D CAD cranial reconstruction with an FFF 3D-printed PEEK PSI.

Computer-assisted conformance assessments were computed using reconstruction contour conformance distance maps. Briefly, the printed cranial implant and 3D CAD cranial defect model surfaces are represented by triangular meshes. After the superimposition and optimal co-registration protocol described in Section 2.3, the distance between each vertex point on the 3D CAD cranial reconstruction with printed PSI (base entity) and the nearest vertex points on the 3D CAD cranial reconstruction with planned PSI (target entity) was computed. The resultant conformance distance was presented in a color-coded map, referred to herein as an overall contour conformance map. For each comparison, the RMSE conformance value was calculated as a metric for morphologic similarity, as described in prior studies [63,64]. The RMSE value provided an estimation of how far the deviation was from zero, and the aberrance of a corresponding data group was represented using a single scale. In addition, the morphological fit and contour continuity of the printed PSI with the 3D CAD cranial defect model was also examined in cross-sectional (axial, coronal, and sagittal) views. This evaluation was further quantified into three distinct criteria: (A) good—complete overlap and tangential fit maintenance around the cranial bone defect margins; (B) satisfactory—partial overlap and tangential fit maintenance around the cranial bone defect margins but acceptable from a clinical point of view; and (C) poor—no overlap and tangential fit maintenance around the cranial bone defect margins and clinically unacceptable. The percentage distribution in subjective evaluation criteria was measured for each morphological fit and contour continuity analysis.

### 4.5. Biomechanical Characterization Protocol for the 3D-Printed PEEK Patient-Specific Cranial Implants

**Figure 7:**
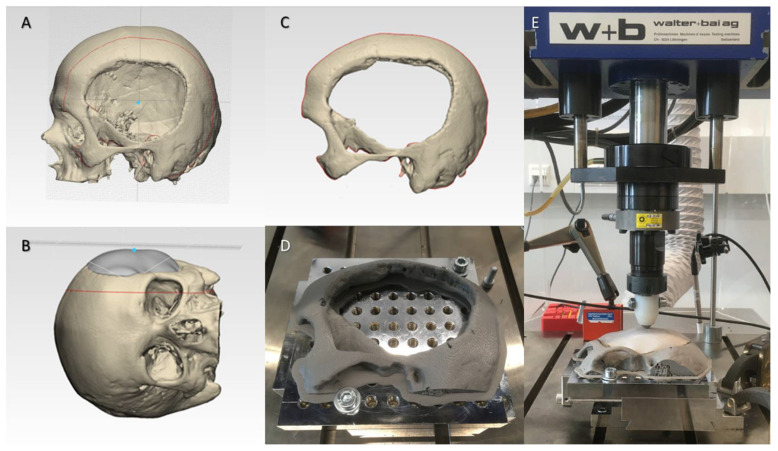
Schematic representation of the steps involved for the biomechanical characterization: (**A**) Computer-aided design (CAD) of a skull model highlighting the centroid mark (blue dot); (**B**) Orientation of the cranial implant along the cartesian axes; (**C**) Computer-aided design (CAD) of the skull template; (**D**) Material extrusion 3D-printed skull template; (**E**) Quasi-static test setup in a servohydraulic system.

The biomechanical properties of the fabricated FFF 3D-printed PEEK cranial implants were accessed in a quasi-static mechanical test setup. A skull template, acting as an underneath support component, was fabricated to allow uniform distribution of the loading force and maintain boundary conditions during the test. Figure 7A shows a skull 3D CAD model emphasizing the centroid on the outer surface. To align the implant along the three coordinate axes, a point on the skull’s normal vector direction was used as a reference focal point (Figure 7B). Once the orientation of the implant along the cartesian axes was assessed, a parallel cutting plane perpendicular to the center point of the implant was performed (Figure 7C). The STL file of the virtually designed skull template was imported into the slicing software (MakerBot Print v. 4.10.0.2046, MakerBot Industries, Brooklyn, New York, NY, USA) of a material extrusion 3D printer (MakerBot Replicator+, MakerBot Industries, Brooklyn, New York, NY, USA). The skull template was printed in polylactic acid (PLA) filament (MakerBot PLA Filament (grey), MakerBot Industries, Brooklyn, New York, NY, USA) and mounted to the base plate of the testing machine (Figure 7D).

The skull template and the implant specimen were placed in a servohydraulic testing machine (Walter + Bai AG Servohydraulic System, Loehningen, Switzerland). The quasi-static setup is illustrated in Figure 7E. A hemispherical indenter (Ø = 10 mm) was used to apply uniaxial compressive load on the PEEK cranial prosthesis, and a load to failure test was conducted. The cranial implant specimens were statically loaded at a constant speed of 1 mm/min in air. A load cell with a capacity of 5 kN was used, and the load was applied at the center of the implant.

After the test, the broken implant or debris was collected, and implants were investigated for failure analysis. We grouped the implants into three classes according to the fracture patterns: class 1 included multi-layered intra- and interlayer fractures with fracture pattern connectivity; class 2 included multi-layered intra- and interlayer fractures without pattern connectivity; and class 3 included linear intralayer fracture patterns only. The percentage distribution in classes 1 to 3 was measured for each fracture pattern analysis.

### 4.6. Statistical Analysis

Descriptive statistics, including the mean, standard deviation (SD), median, interquartile ranges defined as the first quartile to third quartile (Q1 to Q3), and the RMSE values, were collected for all material extrusion 3D-printed PEEK cranial implants. Then, to summarize the quantitative attributes and investigate the geometric and morphological characteristics, RMSE values were computed for each set. Additionally, analyses were performed to assess the peak load and displacement at peak load for each set. First, a Shapiro–Wilk test was conducted to assess the normality and the homoscedasticity of the data. If non-normality of the data was noticed, the Kruskal–Wallis test with pairwise Wilcoxon Rank Sum (Mann–Whitney U) post hoc tests adjusted for multiple-testing using a Holm–Bonferroni correction were carried out to identify intergroup differences. If the interaction was significant, a Tukey’s post hoc test was performed. Statistically significant differences were corroborated for a probability value *p* < 0.05. All data were collected and tabulated in Microsoft Excel 2016, and statistical analysis was performed using R statistical software (R Core Team 3.4.1, The R Foundation for Statistical Computing, Vienna, Austria).

## 5. Conclusions

To the best of the authors’ knowledge, this is the first study to assess the potential clinical efficacy of POC-manufactured FFF 3D-printed PEEK cranial PSIs. Concerning the geometric characteristics, the FFF 3D-printed PEEK cranial PSIs had high dimensional accuracy and repeatability. In addition, the cranial implants revealed clinically acceptable morphological fit and contour continuity. From a biomechanical standpoint, the implants had considerable strength for clinical application; however, variability between the implant’s peak load was noticed. Accordingly, the results of this preclinical study are in line with cranial implant expectations; however, specific attributes have scope for further improvements.

## Figures and Tables

**Table 1:** Quantitative assessment for dimensional repeatability of material extrusion 3D-printed PEEK patient-specific cranial implants.

PSI ^1^	Mean RMSE ^2^ ± SD ^3^	Median RMSE (Q1 to Q3)
1	0.143 ± 0.075	0.121 (0.092 to 0.143)
2	0.143 ± 0.090	0.123 (0.080 to 0.153)
3	0.129 ± 0.079	0.115 (0.076 to 0.138)
4	0.133 ± 0.087	0.114 (0.096 to 0.120)
5	0.129 ± 0.072	0.109 (0.083 to 0.120)
6	0.219 ± 0.024	0.227 (0.214 to 0.232)
7	0.259 ± 0.038	0.270 (0.259 to 0.278)
8	0.134 ± 0.069	0.118 (0.103 to 0.130)
9	0.133 ± 0.081	0.113 (0.099 to 0.116)
10	0.124 ± 0.074	0.098 (0.087 to 0.119)

^1^ Patient-specific implant; ^2^ root mean square error; ^3^ standard deviation.

**Table 2:** Dimensional repeatability assessment of material extrusion 3D-printed PEEK patient-specific cranial implants concerning *p*-values of the Tukey–Kramer post hoc test.

	PSI 1	PSI 2	PSI 3	PSI 4	PSI 5	PSI 6	PSI 7	PSI 8	PSI 9
**PSI 2**	1.00								
**PSI 3**	1.00	1.00							
**PSI 4**	1.00	1.00	1.00						
**PSI 5**	1.00	1.00	1.00	1.00					
**PSI 6**	1.00	1.00	0.51	1.00	0.51				
**PSI 7**	0.29	0.29	0.16	0.22	0.11	0.11			
**PSI 8**	1.00	1.00	1.00	1.00	1.00	0.51	0.03 ^a^		
**PSI 9**	1.00	1.00	1.00	1.00	1.00	1.00	0.22	1.00	
**PSI 10**	1.00	1.00	1.00	1.00	1.00	0.51	0.05 ^b^	1.00	1.00

^a,b^ Within a row, *p*-values with a common superscript letter indicate a statistically significant difference (*p* < 0.05) between the indicated groups. PSI—patient-specific implant.

**Table 3:** Peak force (N) vs. displacement at peak force (mm) in material extrusion 3D-printed PEEK patient-specific cranial implants.

PSI ^1^	Peak Force (N)	Displacement at Peak Force (mm)
1	877.50	2.74
2	1000.31	2.96
3	732.92	2.34
4	626.51	2.59
5	933.93	2.70
6	522.92	1.79
7	541.10	1.87
8	679.72	2.53
9	786.11	2.14
10	1182.91	3.72

^1^ Patient-specific implant.

**Table 4:** Printing parameters selected for the material extrusion 3D-printed PEEK patient-specific cranial implant.

**Extruder**		**Infill**	
Nozzle Diameter (mm)	0.4	Internal Fill Pattern	Rectilinear
**Temperature**		External Fill Pattern	Rectilinear
Extruder Temperature (°C)	485	Infill Percentage	100%
Airflow Temperature (°C)	130–280	Raster angle	45/−45
**Layer**		**Support**	
Layer Height (mm)	0.15	Support Infill (%)	40
Top Solid Layers	3	Support Pillar Resolution (mm)	4
Bottom Solid Layer	3	**Speed (mm/min)**	
Outline/Perimeter Shells	2	Printing speed	2000

## Data Availability

The original contributions presented in the study are included in the article, further inquiries can be directed to the corresponding author.

## Supplementary Materials

Supplementary materials can be found at https://www.mdpi.com/article/10.3390/ijms22168521/s1.

## Abbreviation

3D	Three-Dimensional
AM	Additive Manufacturing
ASTM	American Society for Testing and Materials
CAD	Computer-Aided Design
CAM	Computer-Aided Manufacturing
CT	Computed Tomography
DfAM	Design for Additive Manufacturing
DICOM	Digital Imaging and Communications in Medicine
DSC	Dice Similarity Coefficient
EBM	Electron Beam Melting
FFF	Fused Filament Fabrication
HU	Hounsfield Units
ICP	Iterative Closest Point
ISO	International Organization for Standardization
PLA	Polylactic Acid
PEEK	Polyetheretherketone
POC	Point-of-Care
PSIs	Patient-Specific Implants
RSI	Reconstruction Symmetry Index
RMSE	Root Mean Square Error
SD	Standard Deviation
SLS	Selective Laser Sintering
STL	Standard Tessellation Language
VSP	Virtual Surgical Planning
