# Supplementary material for: Quantitative Assessment of Point-of-Care 3D-Printed Patient-Specific Polyetheretherketone (PEEK) Cranial Implants
Source: Int J Mol Sci. 2021 Aug 7;22(16):8521. doi: 10.3390/ijms22168521 (PMC8395180; doi:10.3390/ijms22168521)
Supplement: Supplementary file 1 [file ijms-22-08521-s001.zip › ijms-1315158-supplementary.pdf]

International Journal of
Molecular Sciences

MDPI

Supplementary Materials

Table S1. Overview of the quasi-static biomechanical test setups for curvilinear-shaped cranial implants.

Author, Year	Material tested	Implant specimen dimensions	Test conditions	Peak load
Our study	PEEK – Material extrusion 3D Printed	***Shape***: patient-specific (complex)<br><br>***Size***: 105 cm^2<br><br>***Thickness***: 4 mm	***Loading rate***: 1 mm/min<br><br>***Indenter***: hemispherical, Ø = 10 mm<br><br>***Support construct***: 3D printed PLA skull model	798.38 ± 211.45 N
Berretta et al., 2018 [41]	PEEK – Laser sintered 3D Printed	***Shape***: patient-specific (complex)<br><br>***Size***: N/A<br><br>***Thickness***: N/A	***Loading rate***: 1 mm/min<br><br>***Indenter***: hemispherical, Ø = 10 mm<br><br>***Support construct***: resin and epoxy adhesive	600-900 N
Lethaus et al., 2011 [46]	PEEK -CAD/CAM milled	***Shape***: simplified<br><br>***Size***: 100 cm^2<br><br>***Thickness***: 6 mm	***Loading rate***: 1.9 mm/min<br><br>***Indenter***: N/A<br><br>***Support construct***: screw-fixated implant on the polyamide skull model	24 kN
Lethaus et al., 2011 [46]	Titanium	***Shape***: simplified<br><br>***Size***: 100 cm^2<br><br>***Thickness***: 6 mm	***Loading rate***: 1.9 mm/min<br><br>***Indenter***: N/A<br><br>***Support construct***: screw-fixated implant on the polyamide skull model	> 50 kN
Ono et al., 1998 [50]	Hydroxyapatite	***Shape***: patient-specific (complex) and simplified	***Loading rate***: 0.5 mm/min<br><br>***Indenter***: N/A<br><br>***Support construct***: N/A	108-225 N

		Size: 113-120 mm x 63-75 mm x 30-38 mm<br>***Thickness***: 6-8 mm		
Stefini et al., 2015 [**51**]	Hydroxyapatite	***Shape***: patient-specific (complex)<br>***Size***: 130-171 cm^2<br>***Thickness***: N/A	***Loading rate***: 1 mm/min<br>***Indenter***: silicone rubber flat, Ø = 25 mm<br>***Support construct***: plaster skull model and epoxy adhesive	586 ± 356 N
Piitulainen et al., 2017 [**52**]	Bioactive glass fiber-reinforced composite	***Shape***: simplified<br>***Size***: 112 x 67 mm^2<br>***Thickness***: 2.5 mm	***Loading rate***: 1 mm/min<br>***Indenter***: rectangular flat (17 x 55 mm)<br>***Support construct***: screw-fixated implant on the aluminum jig	175 ± 101 N
Linder et al., 2019 [**53**]	Titanium-reinforced calcium phosphate (Laser sintered 3D printed Ti mesh)	***Shape***: patient-specific (complex)<br>***Size***: 200 cm^2<br>***Thickness***: 6 mm	***Loading rate***: 1 mm/min<br>***Indenter***: hemispherical, Ø = 10 mm<br>***Support construct***: screw-fixated implant on 3D printed PLA skull model	546 N
Linder et al., 2019 [**53**]	Titanium mesh (Laser sintered 3D printed)	***Shape***: patient-specific (complex)<br>***Size***: 200 cm^2<br>***Thickness***: 1.6 mm	***Loading rate***: 1 mm/min<br>***Indenter***: hemispherical, Ø = 10 mm<br>***Support construct***: 3D printed PLA skull model	479 N
Lewin et al., 2020 [**54**]	Titanium-reinforced calcium phosphate (Laser	***Shape***: simplified<br>***Size***: 80 mm	***Loading rate***: 1 mm/min<br>***Indenter***: hemispherical, Ø = 40 mm	808 ± 29 N

	sintered 3D printed Ti mesh)	***Thickness***: 6 mm	***Support construct***: Stainless steel and calcium phosphate cement with 5 mm silicone rubber surrogate	
Lewin et al., 2021 [**55**]	Titanium-reinforced calcium phosphate (EBM 3D printed Ti mesh)	***Shape***: simplified<br>***Size***: 80 mm<br>***Thickness***: 2.5 mm	***Loading rate***: 1 mm/min<br>***Indenter***: hemispherical, Ø = 40 mm<br>***Support construct***: Stainless steel and calcium phosphate cement with 5 mm silicone rubber surrogate	457 ± 9 N
